# Clinical and Molecular Characterization of Achromatopsia Patients: A Longitudinal Study

**DOI:** 10.3390/ijms22041681

**Published:** 2021-02-07

**Authors:** Raffaella Brunetti-Pierri, Marianthi Karali, Paolo Melillo, Valentina Di Iorio, Antonella De Benedictis, Gennarfrancesco Iaccarino, Francesco Testa, Sandro Banfi, Francesca Simonelli

**Affiliations:** 1 Eye Clinic, Multidisciplinary Department of Medical, Surgical and Dental Sciences, Università degli Studi della Campania “Luigi Vanvitelli”, via Pansini 5, 80131 Naples, Italy; raffaella.brunettipierri@unicampania.it (R.B.-P.); karali@tigem.it (M.K.); paolo.melillo@unicampania.it (P.M.); valentina.diiorio@unicampania.it (V.D.I.); antonella_db@libero.it (A.D.B.); gennarfrancesco.iaccarino@unicampania.it (G.I.); francesco.testa@unicampania.it (F.T.); 2 Telethon Institute of Genetics and Medicine, via Campi Flegrei 34, 80078 Pozzuoli, Italy; banfi@tigem.it; 3 Medical Genetics, Department of Precision Medicine, Università degli Studi della Campania “Luigi Vanvitelli”, via Luigi De Crecchio 7, 80138 Naples, Italy

**Keywords:** achromatopsia, cone photoreceptors, Italian cohort, *CNGA3*, *CNGB3*, *GNAT2*

## Abstract

Achromatopsia (ACHM) is a rare genetic disorder of infantile onset affecting cone photoreceptors. To determine the extent of progressive retinal changes in achromatopsia, we performed a detailed longitudinal phenotyping and genetic characterization of an Italian cohort comprising 21 ACHM patients (17 unrelated families). Molecular genetic testing identified biallelic pathogenic mutations in known ACHM genes, including four novel variants. At baseline, the patients presented a reduced best corrected visual acuity (BCVA), reduced macular sensitivity (MS), normal dark-adapted electroretinogram (ERG) responses and undetectable or severely reduced light-adapted ERG. The longitudinal analysis of 16 patients (mean follow-up: 5.4 ± 1.0 years) showed a significant decline of BCVA (0.012 logMAR/year) and MS (−0.16 dB/year). Light-adapted and flicker ERG responses decreased below noise level in three and two patients, respectively. Only two patients (12.5%) progressed to a worst OCT grading during the follow-up. Our findings corroborate the notion that ACHM is a progressive disease in terms of BCVA, MS and ERG responses, and affects slowly the structural integrity of the retina. These observations can serve towards the development of guidelines for patient selection and intervention timing in forthcoming gene replacement therapies.

## 1. Introduction

Complete achromatopsia (ACHM) (also termed total color blindness or rod monochromacy) is a rare inherited autosomal recessive disease with an estimated prevalence of approximately 1 in 30,000–50,000 worldwide [1]. It has been traditionally classified as a cone dysfunction syndrome rather than a cone dystrophy [2] to reflect its predominantly stationary nature. Nevertheless, there is also growing evidence on the occurrence of progressive morphological and functional retinal changes in this condition [3,4,5]. ACHM has an early age of onset and generally manifests itself with pendular nystagmus at birth or early infancy as a first sign. Affected individuals present photophobia, eccentric fixation, reduced best corrected visual acuity (BCVA) since birth, reduction or complete loss of color perception along the three axes of color vision and small central scotomas [6]. A broad range of refractive errors has been reported in ACHM patients, with hyperopic refraction being the most frequent [7]. The fundus appearance is often normal, but abnormal foveal reflex, pigmentary mottling and atrophic changes may be observed in the macula [5]. In terms of retinal activity, patients show markedly decreased or absent photopic responses (including the 30 Hz flicker response) in full-field electroretinograms (ERG), whereas scotopic responses are normal or only slightly abnormal [8]. The structure of the central retina can be compromised with variable degrees of foveal hypoplasia, disruption and/or loss of the photoreceptors’ ellipsoid zone (EZ band) as well as an attenuation of the retinal pigment epithelium (RPE) at the macula as revealed by optical coherence tomography (OCT) [9]. Fundus autofluorescence imaging (FAF) shows different extents of foveal hypofluorescence. In some cases, a hyper-autofluorescent ring with a central region devoid of autofluorescence (AF) was also reported [3].

There are two clinical forms of ACHM: the complete and incomplete subtypes. Unlike complete achromatopsia, in which cone function is totally abolished leading to profound color blindness, in the incomplete forms the retina retains some residual cone photoreceptor activity as not all three cone spectral types are impaired. These incomplete forms, also termed atypical achromatopsia or dyschromatopsia, are characterized by detectable cone-mediated responses in ERG, a higher BCVA (up to 20/80) and a better-preserved color vision [10].

Pathogenic variants in six genes encoding components of the cone phototransduction cascade (*CNGA3*, *CNGB3*, *PDE6C*, *PDE6H*, *GNAT2*) and of the unfolded protein response (*ATF6*) have been implicated in ACHM [11,12,13,14,15,16,17,18]. Collectively, these genes account for over 90% of ACHM cases, while *CNGA3* and *CNGB3* alone are responsible for 69% of cases [19]. Gene therapy trials are ongoing to correct the defects of *CNGA3* and *CNGB3* that encode, respectively, the α- and β-subunits of the cone photoreceptor cyclic nucleotide-gated ion channel (NCT02599922, NCT03001310, NCT02610582, NCT02935517, NCT03758404) [20,21].

ACHM serves as the ideal target for gene therapies directed to cone photoreceptors, because cones are primarily affected in this disease. Defining the disease course can have important implications for therapies, hence the need to conduct longitudinal studies. Although several studies [4,5,9,22,23,24,25] looked into the clinical course of ACHM patients to assess disease progression, there is still an open debate on whether ACHM is a progressive or stable condition. In particular, some reports showed that at least a subset of patients present progressive vision loss with impaired cone photoreceptor function and poorly preserved foveal integrity [4,5,9,26,27]. In this study, we performed a detailed longitudinal phenotyping and genetic characterization of an Italian cohort of ACHM patients. We describe in depth the clinical features and disease progression in ACHM and investigate possible genotype–phenotype correlations. Our findings, together with similar studies, can serve as a baseline to identify key elements of retinal structure and function in ACHM that can optimize patient selection and intervention timing in forthcoming gene replacement therapies for this condition.

## 2. Results

### 2.1. Patient Selection

The study cohort consisted of 21 patients (mean age at the study baseline: 18.0 ± 3.2 years) (Table S1) from 17 unrelated families. The cohort included three sibling pairs and an uncle–nephew pair. All patients had a clinical diagnosis of ACHM. The main baseline clinical findings are summarized in Table 1. Specifically, 14 patients (66.7%) presented complete ACHM and seven patients (33.3%) had an incomplete form. All patients reported photophobia and 19 patients (90.5%) had congenital nystagmus.

### 2.2. Ophthalmological Assessment of the ACHM Patients

The mean spherical equivalent was +0.16 ± 0.63 D (ranging from −9.0 D to +5 D) (Table S1). The most common refractive error was hyperopia, observed in nine patients (42.9%), whereas myopia was found only in three patients (14.3%). In terms of visual acuity, the mean BCVA at the study baseline was 0.88 ± 0.06 logMAR in both eyes (Table S1) and was generally symmetrical between the two eyes (mean inter-eye asymmetry: 0.04 ± 0.02 logMAR). Only two patients (9.5%) showed values of inter-eye asymmetry equal or higher than the established threshold for clinically significant changes (i.e., 0.3 logMAR). There was no significant correlation between BCVA and age (*p* = 0.102).

**Figure 1:**
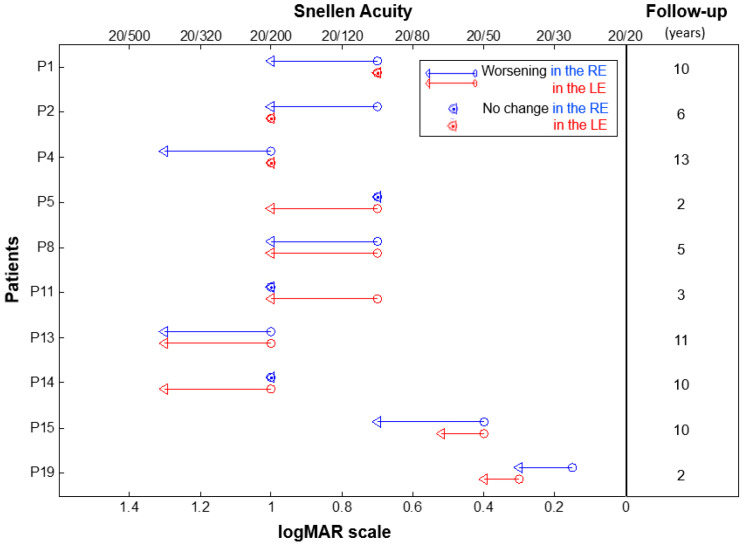
Change in the best corrected visual acuity in individual ACHM patients showing a worsening over the follow-up period. Baseline and last time-point visual acuities are represented by circles and triangles, respectively. Data from the right eye (RE) are in red and left eye (LE) in blue. The duration of the follow-up period (in years) for each patient is shown on the right. Patient ID is shown on the left.

The longitudinal analysis of 16 patients over a mean follow-up of 5.4 ± 1.0 years showed a statistically significant decline of BCVA at a mean annual rate of 0.012 logMAR/year (95% confidence interval (CI): 0.007 to 0.018; standard error (SE): 0.003; *p* < 0.001). During the follow-up, BCVA decreased in 10 out of 16 patients (62.5%). In particular, BCVA worsened bilaterally in four patients (25.0%), whereas six patients (37.5%) presented a unilateral worsening (Figure 1). At the last observation (mean age: 23.5 ± 2.7 years), the mean BCVA was 0.97 ± 0.05 logMAR in both eyes.

All patients performed the Ishihara Pseudoisochromatic Plate test, while 14 patients were also administered the Farnsworth D-15 color test. The Ishihara color vision test ascertained the inability of all patients to read any pseudoisochromatic plate apart from the test plate. The Farnsworth D-15 color test revealed a complete loss of color perception along all three axes of color vision in 12 patients, whereas one patient (P19) was classified as having deuteranopia and tritanopia. One patient (P18) failed to perform the Farnsworth D-15 test due to low BCVA and poor collaboration (Table 1)**.**

**Figure 2:**
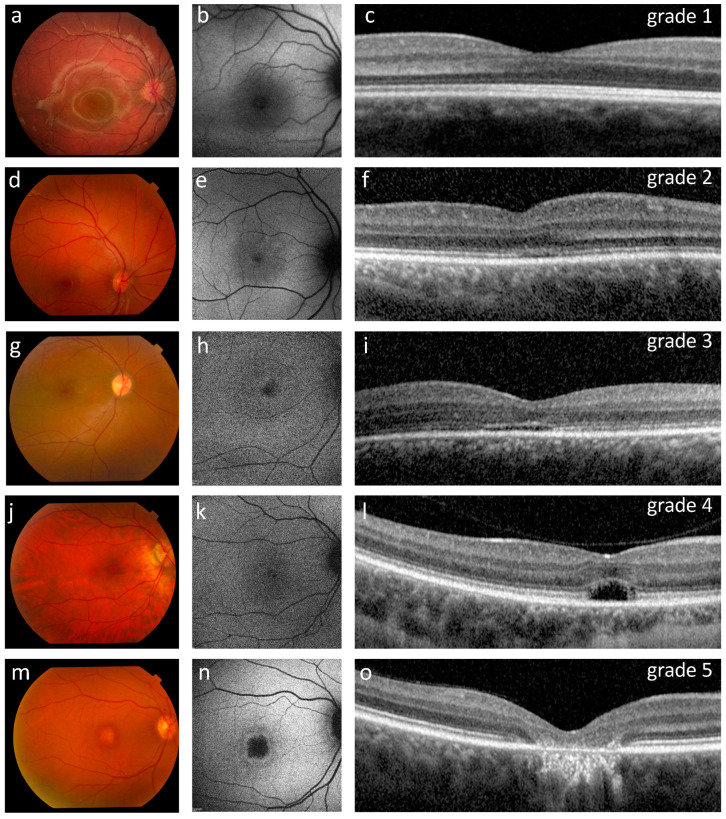
Ophthalmological findings in five representative ACHM patients showing the OCT grading (**c**,**f**,**i**,**l**,**o**) with corresponding retinography (**a**,**d**,**g**,**j**,**m**) and fundus AF images (**b**,**e**,**h**,**k**,**n**). (**a**–**c**) Patient P15 (12 years old, homozygous for the c.619G>A variant in *GNAT2*) presented a normal fundus appearance, normal fundus autofluorescence and a grade 1 OCT with a continuous ellipsoid zone (EZ band). (**d**–**f**) Patient P21 (16 years old) had a normal appearing fundus, reduced autofluorescence with subtle hyper-autofluorescence around the nasal fovea, and a grade 2 OCT with a disrupted EZ band. (**g**–**i**) Patient P19 (19 years old) presented a normal fundus, foveal and parafoveal hyper-autofluorescence, and a grade 3 OCT characterized by an absent EZ band. (**j**–**l**) Patient P6 (32 years old, homozygous for the c.847C>T variant in *CNGA3*) had a normal fundus appearance, foveal and parafoveal hyper-autofluorescence, and a grade 4 OCT characterized by the presence of a hypo-reflective zone. (**m**–**o**) Patient P10 (46 years old, homozygous for the c.1148delC variant in *CNGB3*) presented retinal pigment epithelium (RPE) dystrophy, a central region devoid of autofluorescence surrounded by a hyper-autofluorescent ring, and a grade 5 OCT characterized by outer retinal atrophy including RPE loss.

All patients had clear optic media (cornea and lens) and a normal appearing fundus (Figure 2), except for one case (P10) who had RPE macular dystrophy in both eyes (Figure 2m).

### 2.3. Retinal Structure in ACHM Patients

At the baseline OCT, nine patients (42.9%) had a continuous EZ band in both eyes (foveal structure of grade 1) (Figure 2c), five (23.8%) had an EZ band disruption (grade 2) (Figure 2f), one patient (4.8%) had an absent EZ band (grade 3) (Figure 2i), three patients (14.3%) had a hyporeflective zone (HRZ) (grade 4) (Figure 2l), and one patient (4.8%) showed outer retinal atrophy including RPE loss (grade 5) in both eyes (Figure 2o). Representative examples of each OCT grade are shown in Figure 2. Moreover, two patients had a HRZ in one eye and either an EZ band disruption (4.8%) or an absent EZ band in the contralateral eye (4.8%) (Table 1). Significant differences (ANOVA, *p* = 0.02) were observed in the age of the patients stratified according to the OCT classification, when considering the groups that comprised at least three cases (i.e., grades 1, 2, 4). In particular, grade 4 patients were significantly older (35.3 ± 2.6 years) than grade 1 (11.0 ± 8.6 years; *p* = 0.006) and grade 2 (18.0 ± 7.8 years; *p* = 0.048).

Mean central retinal thickness (CRT) was 224.4 ± 9.8 µm in the right eyes and 213.9 ± 8.2 µm in the left eyes (Table S1). Only three patients (14.3%) showed foveal hypoplasia bilaterally (Table 1). Cross-sectional analysis showed that CRT significantly decreased with patient’s age at baseline (β: −1.3; SE: 0.6; *p* = 0.042), whereas the longitudinal data did not show significant changes in CRT during the follow-up period (*p* = 0.993). Furthermore, in terms of OCT grading, only two patients (12.5%) progressed to a worse OCT stage over the follow-up: one patient from grade 2 to 3 in one eye only (P8; left eye), the other from grade 1 to 2 in both eyes (P2) (Figure S1).

FAF imaging (available for 14 patients) revealed significant abnormalities in 11 subjects: one patient (7.1%) showed a central region of absent AF with a surrounding hyper-autofluorescent ring, three patients (21.4%) presented a reduced AF with subtle hyper-autofluorescence around the fovea, three patients (21.4%) had widespread reduced AF, and four patients (28.6%) showed foveal and parafoveal hyper-autofluorescence (Table 1). We also observed that the patient with a grade 5 OCT had a central region devoid of AF surrounded by a hyper-autofluorescent ring (Figure 2n). OCT grades 3 and 4 were associated with foveal and parafoveal hyper-autofluorescence (three out of five cases) (Figure 2h,k). On the other hand, patients belonging to OCT grades 1 and 2 (eight cases) had variable distributions of fundus autofluorescence that included a reduced AF with subtle hyper-autofluorescence around the fovea (two out of eight cases) (Figure 2e), a foveal and parafoveal hyperautofluorescence (one out of eight cases), a widespread reduced AF (two out of eight cases) or a normal AF (three out of eight cases) (Figure 2b, Table 1).

### 2.4. Retinal Function in the ACHM Patients

Microperimetric assessment (available for 18 patients) showed a reduced mean macular sensitivity (MS) in both eyes (14.6 ± 0.9 dB in right eyes, 15.2 ± 0.9 dB in left eyes) at the baseline (Table S1). MS decreased significantly with patient’s age (β = −0.312 dB/year; SE: 0.09; *p* = 0.001). It declined further during the follow-up period (5.4 ± 1.0 years) at a mean annual rate of −0.16 dB/year (SE: 0.05; *p* = 0.003). In terms of fixation stability, one patient (5.6%) showed stable fixation in both eyes, seven (38.9%) had a relatively stable fixation and two (11.1%) patients had unstable fixation in both eyes. The remaining patients (44.4%) showed inter-ocular asymmetry in the fixation stability: stable vs. relatively stable fixation (one patient) and relatively stable vs. unstable fixation (seven patients) (Table 1).

At the baseline ERG assessment which was performed for the entire cohort (n = 21), all patients showed normal dark-adapted 0.01 ERG responses (mean b-wave amplitude: 147.2 ± 11.7 µV in the right eyes; 163.0 ± 10.9 µV in the left eyes) (Table S1). On the contrary, light-adapted 3.0 ERG responses were undetectable in most patients (15/21; 71.4%) (Table 1). Only six patients (28.6%) had detectable light-adapted 3.0 ERG (mean age: 13.1 ± 2.7 years) with a mean b-wave amplitude of 12.2 ± 3.5 µV in the right eyes and 14.7 ± 7.4 µV in the left eyes (Table S1). Similarly, flicker ERG responses were extremely reduced, yet detectable, in the majority of patients (14/21; 66.7%) (Table 1), with a mean N1-P1 amplitude of 5.1 ± 1.2 µV in the right eyes and of 6.0 ± 1.4 µV in the left eyes (Table S1). Moreover, detectable light-adapted 3.0 responses associated with a significantly higher CRT (β = 18.9 µm; *p* = 0.031), higher MS (β = 6.6 dB; *p* < 0.001), and with higher values of b-wave amplitudes in the dark-adapted response (β = 61.9 µV; *p* = 0.004). Finally, detectable flicker responses correlated with a better BCVA (β = −0.46 logMAR; *p* = 0.002) and higher MS (β = 4.6 dB; *p* = 0.007).

Longitudinal analysis (mean follow-up: 5.4 ± 0.9 years) did not show a significant reduction neither of the b-wave amplitude in dark- (−0.6 µV per year; *p* = 0.624) and light-adapted 3.0 ERG responses (−0.3 µV per year; *p* = 0.318) nor of the N1-P1 amplitude in flicker ERG responses (−0.4 µV per year; *p* = 0.084). However, we observed a reduction of light-adapted 3.0 ERG below noise level in three (P1, P9, P12) out of the six patients, and reduction below noise level of flicker ERG in only two patients out of 14 (P8, P9).

### 2.5. Genetic Analysis of ACHM Patients

To determine the genetic defect underlying their condition, we performed high-throughput sequencing analysis of patients who gave consent to genetic testing (n = 18; P19–P21 did not consent). These samples were analyzed either by panel-based approaches or by clinical exome sequencing. One case (P18) that remained unsolved after a panel-based analysis of 130 retinopathy genes (RETplex) was subsequently analyzed by whole-exome sequencing (WES).

Overall, we identified causative variants that could explain the clinical phenotype in 16 ACHM patients (from 13 unrelated families) (Table 2). These patients harbored biallelic variants in one of the six genes that are more commonly associated with ACHM. Specifically, seven patients had biallelic mutations in *CNGA3*, five patients in *CNGB3*, three in *GNAT2*, and one in *PDE6C* (Table 2). Two patients with typical ACHM remained unsolved. They included a female patient (P17) with a monoallelic missense variant of unknown significance (c.1055G>A; p.Arg352Lys) in *CNGB3* that alone cannot explain the clinical phenotype, and a male patient (P18) analyzed by WES following a negative first-tier screening using the RETplex panel.

A third of the causative variants identified in the cohort (i.e., 4 out of 12) were novel and absent from reference databases (e.g., HGMD, LOVD). One variant (*GNAT2*, c.832dup) was predicted to generate a frameshift leading to a premature stop codon. The remaining mutations were missense variants of uncertain significance according to the classification of the American College of Medical Genetics, predicted in silico to be deleterious for the protein function (Table 3).

### 2.6. Genotype-Phenotype Correlation Analysis

To explore possible genotype–phenotype correlations, we sought for differences in the clinical presentation between the patients with mutations in the *GNAT2*, *CNGB3* and *CNGA3* genes. There were no significant differences in the selected clinical parameters among the three genotypic groups (Table 4). Nonetheless, we observed a higher frequency of detectable flicker ERG responses in *CNGB3*-ACHM (80%) and *GNAT2*-ACHM patients (100%) compared to *CNGA3*-ACHM patients (28.6%) (Table 4).

## 3. Discussion

Here we provide the first detailed clinical description and molecular characterization of an Italian cohort of ACHM patients. To the best of our knowledge, this is one of the longest longitudinal studies of ACHM patients with a mean follow-up period of more than 5 years. Delineating the clinical features and the natural history of ACHM is both relevant and timely, given that phase I/II gene supplementation trials aimed at patients with causative mutations in *CNGA3* and *CNGB3* are ongoing [20,21].

Our results suggest that ACHM is characterized by a progressive decline in visual function and a slow deterioration of the macular structure. First, we found that BCVA worsened significantly in 62.5% of our patients during a follow-up period of approximately 5 years. Previous studies reported a progressive decline in BCVA in a smaller percentage of cases (about 10%) [9,31], but these discrepancies may be related to differences in the follow-up length or in the frequency of mutated genes among the cohorts. Second, our findings indicate that the integrity of retinal structure is overall well preserved during the course of the disease within the time-frame studied. In particular, CRT did not change significantly during the follow-up period and only two patients (12.5%) progressed to a more severe OCT grade. Along the same line, other studies reported a progressive loss of foveal integrity in a subset of ACHM patients (about 5–10%) over a mean follow-up between 2 and 5 years [3,4,5,9,22]. However, we found that older patients had a lower CRT and a more advanced OCT grade (i.e., grade 4 patients were mainly in the fourth decade of life, whereas grade 1 and grade 2 patients were mainly in the first two decades) suggesting that changes in retinal structure occur slowly, for instance over decades of life. Therefore, monitoring patients for periods longer than 5 years may reveal progressive changes in a greater proportion of individuals. Moreover, extended follow-up periods may also enable to assess whether the aging of the retina progresses at a different speed in patients with achromatopsia.

As regards the retinal function, we found that MS negatively correlated with patient’s age and decreased significantly over the follow-up period. To date, only few studies assessed the MS of ACHM patients by MP [9,23,24,32]. Our findings are in agreement with the observations of Sundram et al. who reported a decline of MS with age in 40 patients [23]. Conversely, Aboshiha et al. and more recently Georgiou et al. did not observe significant changes in MS in 38 and 18 patients, respectively [9,24]. These discrepancies may be due to the shorter follow-up reported in the first case (i.e., 19.5 months) [9] and to the different distribution of mutated genes [24]. Finally, we observed unrecordable or markedly reduced light-adapted responses that did not change significantly during the follow-up. On the other hand, the amplitude of the photopic ERG decreased below noise level in half of patients with detectable photopic responses at baseline. Likewise, Khan et al. observed that some patients retain low levels of residual cone function into adulthood which is then progressively lost [33]. Taken together, the above functional read-outs suggest that patients with ACHM may have substantial residual cone function despite having a significantly abnormal ERG response and lower MS. In light of these findings, MP could provide useful insights into the retinal function of ACHM patients and guide therapeutic decision-making and patient selection for inclusion in clinical trials.

In our cohort, we identified biallelic pathogenic variants in four of the six genes that are typically implicated in ACHM, namely *CNGA3*, *CNGB3*, *GNAT2*, *PDE6C*. Mutations in *CNGA3* and *CNGB3* accounted for the majority of molecularly defined cases (75%, 12 out of 16 cases), consistent with the high prevalence of *CNGA3* and *CNGB3* mutations in autosomal recessive achromatopsia [28,34]. The most recurrent *CNGB3* variant was the c.1148delC (representing 80% of the identified *CNGB3* alleles), in line with the observed frequency of this allele in other *CNGB3*-ACHM cohorts [13,34,35]. We also identified two novel missense mutations in *CNGA3* (c.1162G>A; p.(Gly388Ser)) and *CNGB3* (c.1055G>A; p.(Arg352Lys)) that are absent from population databases and extend the list of known likely pathogenic variants. Additional novel disease-causing variants were identified in *GNAT2*, which was less frequently mutated in our cohort. The majority of solved cases (81%, 13 out of 16 cases) were homozygous for the identified variants, as reported also in other ACHM cohorts [36].

Two analyzed cases remained unsolved. One subject was heterozygous for a likely pathogenic variant in *CNGB3*. We believe that this finding is not incidental, given the low allele frequency of the identified variant (absent from gnomAD) and the patient’s clinical presentation of typical ACHM. In this case, the missing causative variant could lie in noncoding sequences, such as a deep-intronic splice variant that causes pseudoexon activation in *CNGB3* [35]. Another hypothesis may be the presence of a copy number variation (CNV), which is expected to be undetected by our capture-based targeted NGS screening. *CNGB3* was predicted to be prone to CNVs due to its genomic features and high *Alu* repeat content [37], and complementary targeted CNV screening proved decisive in the analysis of monoallelic *CNGB3* patients [36]. These explanations are valid also for the second case that presented the hallmarks of ACHM but remained unsolved even after WES.

In an attempt to assess whether mutations in the three most prominent genes of the cohort (*CNGA3*, *CNGB3* and *GNAT2*) associate with distinct clinical parameters, we performed a genotype–phenotype correlation analysis. We did not identify statistically significant differences in the disease course and clinical features among the three genotypic groups, which may be attributable to the limited size of the analyzed cohort. Since it was hypothesized that foveal hypoplasia may represent a gene-specific feature [38] (as it was observed in the majority of previously reported *CNGA3*- and *CNGB3*-ACHM subjects but not in patients carrying defects in *GNAT2* or *PDE6C* [38]), we looked into this feature in relation to the patient’s genotype. In our cohort, foveal hypoplasia was not observed in *GNAT2-* and *PDE6C*-ACHM patients while it was observed only in a subgroup of patients harboring pathogenic variants in *CNGB3* and *CNGA3*, although at a much lower frequency (about 17%).

This study has some limitations in its retrospective design. First, although the patients were invited to undergo annual follow-up visits, the follow-up length was not the same for all patients and only cross-sectional data were available for some patients or some tests. Moreover, microperimetric measurements and FAF imaging were available only for a subset of the cohort because some patients were not always willing or capable to perform the examination (i.e., because of poor BCVA, young age). Finally, we acknowledge that the relatively small sample size may hinder the detection of statistically significant associations in some comparisons (i.e., groups by genotype) due to the insufficient power of the test.

In conclusion, a better understanding of the disease course and progression are crucial for patient management, with important implications for therapeutic considerations. Our findings sustain that ACHM is a progressive disease in terms of BCVA, MS and ERG responses and affects slowly the structural integrity of the retina. In particular, MP could be useful in ACHM as an informative test to guide therapeutic decisions and establish appropriate timing for interventions. Ultimately, our study can contribute towards the development of guidelines for patient selection and intervention timing in forthcoming gene replacement therapies.

## 4. Materials and Methods

### 4.1. Ethics Statement

The retrospective observational study was performed at the Referral Centre for Inherited Retinopathies of the Eye Clinic, Multidisciplinary Department of Medical, Surgical and Dental Sciences, University of Campania “Luigi Vanvitelli”. All procedures of this study adhered to the tenets of the Declaration of Helsinki and were approved by the Ethics Board of the Università degli Studi della Campania “Luigi Vanvitelli” (for adult protocol n. 8189/2015, 9 April 2015; for pediatric subjects’ protocol n. 500/2017, 12 September 2017). Peripheral blood samples were collected upon written informed consent of the patients to sample collection and genetic analysis. For minors, informed consent was obtained by the parents or legal guardians.

### 4.2. Patients Inclusion Criteria

All patients evaluated at the Referral Centre for Inherited Retinopathies of the Eye Clinic of the University of Campania “Luigi Vanvitelli” with a clinical diagnosis of ACHM were included in the study. The clinical diagnosis of ACHM was performed according to previously established criteria [39]. Specifically, the diagnostic criteria included pendular nystagmus, photophobia, reduced BCVA (varies from 20/200 or less in complete ACHM and may be as high as 20/80 in incomplete ACHM), reduction (incomplete ACHM) or complete loss (complete ACHM) of color perception along all three axes of color vision, normal fundus appearance in many affected patients (although absence of the foveal reflex, pigment mottling or atrophy in the macular region may occur in older individuals), and absent (complete ACHM) or residual (incomplete ACHM) cone responses with normal rod responses in ERG.

### 4.3. Ophthalmological Examination

The patient’s medical history was obtained prior to examination. All patients underwent a full ophthalmological examination which included BCVA measurements, color vision testing, slit lamp anterior segment examination, fundus examination, OCT, FAF, MP, and ERG. For 16 subjects, follow-up measurements of BCVA, OCT, MP, and ERG were available for a mean period of 5.4 ± 1.0 years.

Color vision testing was carried out using the Pseudoisochromatic Plate Ishihara and/or the Farnsworth D-15 test.

Based on the fundus appearance, each patient was classified into one of the following categories: (i) normal (no RPE disturbance), (ii) RPE macular dystrophy, or (iii) RPE macular atrophy [9].

OCT was performed on both eyes following pupillary dilation (tropicamide 1% and phenylephrine 2.5% eye drops), using a spectral domain OCT (Spectralis OCT plus with blue peak, Heidelberg Engineering, Heidelberg, Germany) by experienced operators (RBP, VDI, FT). At follow-up visits, the device was switched to the follow-up mode in order to scan the same location both at baseline and in subsequent follow-ups. Qualitative assessment of foveal structure was performed by grading spectral domain OCT images into one of the five distinct categories, according to Aboshiha et al. [9]: continuous EZ band (grade 1), EZ band disruption (grade 2), EZ band absence (grade 3), presence of a HRZ (grade 4), outer retinal atrophy including RPE loss (grade 5). The presence or absence of foveal hypoplasia, defined as the persistence of one or more inner retinal layers (outer plexiform layer, inner nuclear layer, inner plexiform layer or ganglion cell layer) through the fovea, was also assessed [23]. Retinal thickness was calculated manually by identifying the thinnest point in the fovea.

FAF imaging was performed subsequently to the acquisition of the OCT images, due to the relatively intense lights used during FAF acquisition and the photophobic nature of ACHM patients. Pupillary dilatations were performed as described above. Consensus OCT grading and FAF pattern were established by two authors (RBP, VDI). When interpretations differed, a discussion was made to reach an agreement and a third author (FT) also gave his interpretation, if necessary.

MP was performed by an automatic fundus-related perimeter (MP1 Microperimeter, Nidek Technologies, Padova, Italy). The following parameters were used: a fixation target of 2 degrees in diameter consisting of a red ring; a white, monochromatic background with a luminance of 1.27 cd/m²; a Goldmann III-size stimulus with a projection time of 200 ms; a predefined automatic test pattern (Humphrey 10-2) covering 10 degrees centered onto the gravitational center of the fixation points with 68 stimuli [40].

ERG was recorded according to the International Guidelines of the International Society of Clinical Electrophysiology of Vision (ISCEV) [41,42].

### 4.4. Target Enrichment and Next-Generation Sequencing

Samples were analyzed either by panel-based analysis of known retinopathy genes or by clinical exome sequencing. One sample underwent whole-exome sequencing. Genomic DNA was extracted from peripheral blood using the DNeasy Blood and Tissue Kit (QIAGEN) according to standard protocols. Targeted NGS analysis was performed using the RETplex targeted sequencing panel as previously described [30]. Clinical exome and whole-exome sequencing libraries were prepared using the ClearSeq Inherited Disease Panel (Agilent Technologies, Santa Clara, CA, USA) and the SureSelect Human All Exon V7 (Agilent Technologies, Santa Clara, CA, USA), respectively. Targeted regions were enriched using the SureSelectQXT Target Enrichment system (Agilent Technologies, Santa Clara, CA, USA). Libraries were run on a NextSeq500 sequencing platform (Illumina inc., San Diego, CA, USA). Sequencing data were analyzed using a previously described pipeline [43].

### 4.5. Variant Interpretation and Validation

The single nucleotide variants (SNVs) and indel variants were annotated using ANNOVAR [44] with the relative position in genes using the RefSeq87 gene model, amino acid change, presence in dbSNP v137, frequency in the EXAC (http://exac.broadinstitute.org) and gnomAD databases (http://gnomad.broadinstitute.org), 1000 genomes project [45], presence in the Human Gene Mutation Database (HGMD) [46], Clinvar database [47], multiple cross-species conservation [48], and prediction scores of damage on protein activity [49,50,51,52]. Only variants with minor allele frequency (MAF) <0.01 were considered. The alignments at candidate positions were visually inspected using the Integrative Genomics Viewer (IGV).

The identified variants were validated by Sanger sequencing of the corresponding genomic fragments. Amplicons were obtained by PCR using Taq polymerase according to standard protocols and were Sanger sequenced. Mutation detection was performed using the CodonCode Aligner software. Segregation analysis of the identified variants was performed whenever samples from additional family members were available.

### 4.6. Statistical Analysis

Continuous variables are reported as mean ± standard error of mean (SEM) and categorical variables are reported as counts (percentage).

The natural history of disease was analyzed using previously applied methods [53,54]. In order to include all the patients (i.e., also those with no longitudinal data), we performed a cross-sectional analysis on the data of the baseline visit for each of the selected outcome measures (i.e., BCVA, CRT, MS). In particular, linear regression models, estimated by a generalized estimating equation (GEE), were performed with each outcome measure as the dependent variable and age as the independent variable in order to estimate a slope (mean rate of change per year of age) with its relative standard error (SE). Moreover, the GEE was fitted on longitudinal data, using baseline values as offset and follow-up length expressed in years as an independent variable, to estimate the annual change of each outcome over the follow-up period. Finally, regression models were fitted with each outcome measure as the dependent variable and mutated genes (i.e., *CNGA3*, *CNGB3* and *GNAT2*) as independent variable, in order to investigate whether the genotype influenced the selected outcome.

GEE was applied because this method could accommodate the inter-eye correlation (i.e., between the two eyes of the same subject at a given visit) and longitudinal correlation (i.e., between values of the same eye followed over time) by adopting an appropriate covariance structure, as previously described [55]. Intercepts were included in all the models, except those regarding BCVA, since we assume that BCVA should start with the value of 0 logMAR (corresponding to healthy status). Moreover, asymmetry in BCVA between the two eyes was defined as a difference of 0.3 logMAR (15 Early Treatment Diabetic Retinopathy Study (ETDRS) letters), which is the threshold for clinical significance of BCVA changes [56].

Finally, analysis of variance (ANOVA) with Bonferroni correction for multiple comparisons performed with t-tests was adopted to evaluate differences in the age between the patients classified according to OCT grade.

*p*-values (*p*) lower than 0.05 were considered statistically significant. These statistical analyses were performed using the IBM SPSS Statistics platform (Version 21.0.0.0).

## Figures and Tables

**Table 1:** Main clinical findings in each achromatopsia patient at the study baseline.

ID	Age (yrs)	ACHM Subtype	Cong. Nystag. *	Color Vision Test	BCVA ^†^ (RE-LE)		OCT Grade (RE-LE)		CRT ^$^ (RE-LE)		Foveal Hypo-Plasia	FAF Pattern	MS ^$^ (RE-LE)		Fixation Stability (RE-LE)		Dark-Adapted 0.01 ERG	Light-Adapted 3.0 ERG	Light-Adapted 30 Hz ERG
P1	7	INC	No	CB (I)	0.70	0.70	1	1	219	232	Yes	n/a	18.3	18.7	RS	RS	Normal	Reduced	Reduced
P2	9	INC	Yes	CB (F)	0.70	1.00	1	1	265	274	No	Reduced AF, subtle perifoveal hyper-AF	19.6	19.7	UN	RS	Normal	Reduced	Reduced
P3	29	COM	Yes	CB (F)	1.00	1.00	2	2	163	171	No	n/a	13.2	13.9	RS	RS	Normal	Undetectable	Undetectable
P4	18	COM	Yes	CB (F)	1.00	1.00	3	4	167	189	No	Widespread reduced AF	9.8	17.6	S	S	Normal	Undetectable	Undetectable
P5	19	COM	Yes	CB (I)	0.70	0.70	1	1	267	253	No	n/a	12.6	15.4	RS	RS	Normal	Undetectable	Undetectable
P6	32	COM	Yes	CB (F)	1.00	1.00	4	4	239	228	No	Foveal and parafoveal hyper-AF	10	9.7	RS	RS	Normal	Undetectable	Undetectable
P7	59	COM	Yes	CB (F)	1.00	1.00	4	4	187	233	No	Reduced AF, subtle perifoveal hyper-AF	10	10	UN	RS	Normal	Undetectable	Undetectable
P8	26	INC	Yes	CB (F)	0.70	0.70	2	2	235	221	Yes	Widespread reduced AF	16.1	17.8	UN	RS	Normal	Reduced	Reduced
P9	8	INC	Yes	CB (I)	1.00	1.00	1	1	271	267	No	Widespread reduced AF	17	17.8	UN	RS	Normal	Reduced	Reduced
P10	46	COM	Yes	CB (F)	1.30	1.30	5	5	125	115	No	Absent AF at center, hyper-AF ring	9.7	9.3	RS	RS	Normal	Undetectable	Undetectable
P11	10	COM	Yes	CB (F)	1.00	0.70	2	2	244	238	No	n/a	16.8	16.5	UN	RS	Normal	Undetectable	Reduced
P12	4	INC	Yes	CB (F)	1.00	1.00	2	2	227	217	No	n/a	18.5	18.3	RS	UN	Normal	Reduced	Reduced
P13	7	COM	Yes	CB (I)	1.00	1.00	1	1	304	196	No	Foveal and parafoveal hyper-AF	9.3	8.5	RS	UN	Normal	Undetectable	Reduced
P14	5	COM	Yes	CB (I)	1.00	1.00	1	1	258	230	No	n/a	14.4	15	UN	UN	Normal	Undetectable	Reduced
P15	2	INC	Yes	CB (I)	0.40	0.40	1	1	260	166	No	Normal	19.6	19.7	RS	RS	Normal	Reduced	Reduced
P16	15	COM	Yes	CB (F)	0.70	0.70	4	4	196	218	No	Foveal and parafoveal hyper-AF	14.5	14	UN	UN	Normal	Undetectable	Reduced
P17	7	COM	Yes	CB (I)	1.30	1.30	1	1	229	184	No	n/a	n/a	n/a	n/a	n/a	Normal	Undetectable	Reduced
P18	15	COM	Yes	CB (I)	1.00	1.00	2	2	183	206	No	Normal	13.5	13.2	RS	RS	Normal	Undetectable	Reduced
P19	15	INC	No	Deu, Tri (F)	0.15	0.30	3	3	182	187	No	Foveal and parafoveal hyper-AF	19.4	18.7	S	RS	Normal	Undetectable	Reduced
P20	31	COM	Yes	CB (F)	1.00	1.00	1	1	220	207	No	Normal	n/a	n/a	n/a	n/a	Normal	Undetectable	Undetectable
P21	16	COM	Yes	CB (F)	0.70	0.70	2	4	271	259	Yes	Reduced AF, subtle perifoveal hyper-AF	n/a	n/a	n/a	n/a	Normal	Undetectable	Reduced

AF, autofluorescence; BCVA, best corrected visual acuity; CB, color blindness; COM, complete form; CRT, central retinal thickness; Deu, deuteranopia; ERG, electroretinogram; F, Farnsworth D-15 color test; I, Pseudoisochromatic Plate Ishihara; INC, incomplete form; LE, left eye; MS, macular sensitivity; n/a, not available; RE, right eye; RS, relatively stable; S, stable; Tri, tritanopia; UN, unstable; yrs, years. * All patients had photophobia. ^†^ BCVA expressed in logMAR. ^$^ CRT and MS expressed in µm.

**Table 2:** Genetic findings in the achromatopsia patients.

ID	Family	Gene	Zygosity	RefSeq	Allele 1			Allele 2		
					Nucleotide	Protein	Reference	Nucleotide	Protein	Reference
P1	F1	*CNGA3*	Hom.	NM_001298	c.1114C>T	p.Pro372Ser	[28]	c.1114C>T	p.Pro372Ser	[28]
P2	F2	*CNGA3*	Hom.	NM_001298	c.1641C>A	p.Phe547Leu	[16]	c.1641C>A	p.Phe547Leu	[16]
P3	F3	*CNGA3*	Hom.	NM_001298	c.1641C>A	p.Phe547Leu	[16]	c.1641C>A	p.Phe547Leu	[16]
P4	F4	*CNGA3*	Hom.	NM_001298	c.1162G>A	p.(Gly388Ser)	This study	c.1162G>A	p.(Gly388Ser)	This study
P5	F5	*CNGA3*	C. Het.	NM_001298	c.667C>T	p.Arg223Trp	[28]	c.1060T>C	p.Ser341Pro	[28]
P6	F6	*CNGA3*	Hom.	NM_001298	c.847C>T	p.Arg283Trp	[16]	c.847C>T	p.Arg283Trp	[16]
P7	F6	*CNGA3*	Hom.	NM_001298	c.847C>T	p.Arg283Trp	[16]	c.847C>T	p.Arg283Trp	[16]
P8	F7	*CNGB3*	Hom.	NM_019098	c.1148delC	p.(Thr383Ilefs*13)	[29]	c.1148delC	p.(Thr383Ilefs*13)	[29]
P9	F8	*CNGB3*	Hom.	NM_019098	c.1148delC	p.(Thr383Ilefs*13)	[29]	c.1148delC	p.(Thr383Ilefs*13)	[29]
P10	F9	*CNGB3*	Hom.	NM_019098	c.1148delC	p.(Thr383Ilefs*13)	[29]	c.1148delC	p.(Thr383Ilefs*13)	[29]
P11	F10	*CNGB3*	C. Het.	NM_019098	c.1148delC	p.(Thr383Ilefs*13)	[29]	c.970A>G	p.(Arg324Gly)	[30]
P12	F10	*CNGB3*	C. Het.	NM_019098	c.1148delC	p.(Thr383Ilefs*13)	[29]	c.970A>G	p.(Arg324Gly)	[30]
P13	F11	*GNAT2*	Hom.	NM_005272	c.832dup	p.(Ile278Asnfs*14)	This study	c.832dup	p.(Ile278Asnfs*14)	This study
P14	F11	*GNAT2*	Hom.	NM_005272	c.832dup	p.(Ile278Asnfs*14)	This study	c.832dup	p.(Ile278Asnfs*14)	This study
P15	F12	*GNAT2*	Hom.	NM_005272	c.619G>A	p.(Glu207Lys)	This study	c.619G>A	p.(Glu207Lys)	This study
P16	F13	*PDE6C*	Hom.	NM_006204	c.2017G>T	p.(Asp673Tyr)	[30]	c.2017G>T	p.(Asp673Tyr)	[30]
P17	F14	*CNGB3 ?*	Het.	NM_019098	c.1055G>A	p.(Arg352Lys)	This study	n.d.	n.d.	n.d.
P18	F15	n.d.	n.d.	n.d.	n.d.	n.d.	n.d.	n.d.	n.d.	n.d.

C. Het., compound heterozygous; Het., heterozygous; Hom., homozygous; n.d., not determined; ?, uncertain.

**Table 3:** Pathogenicity predictions for the novel missense variants reported in this study.

Gene	RefSeq	Nucleotide	Protein	In Silico Pathogenicity Analysis		
				MutationTaster ^†^	PolyPhen-2 ^‡^	Cadd13 ^#^
*CNGA3*	NM_001298	c.1162G>A	p.(Gly388Ser)	Disease causing	Prob. damaging	26.1
*CNGB3*	NM_019098	c.1055G>A	p.(Arg352Lys)	Disease causing	Prob. damaging	37.0
*GNAT2*	NM_005272	c.619G>A *	p.(Glu207Lys)	Disease causing	Prob. damaging	29.4

^†^http://www.mutationtaster.org/. ^‡^ Polymorphism Phenotyping v2; http://genetics.bwh.harvard.edu/pph2/. ^#^ Combined Annotation-Dependent Depletion; http://cadd.gs.washington.edu/. * present in gnomAD.

**Table 4:** Comparison of ACHM patients stratified according to the genotype.

Parameters	*CNGA3*		*CNGB3*		*GNAT2*		*p*
	(Seven Patients)		(Five Patients)		(Three Patients)		
Age (years)	24.7 ± 6.7		18.7 ± 7.7		4.64 ± 1.6		0.238
Age at diagnosis (years)	5.4 ± 2.8		11.2 ± 18.4		4.6 ± 1.5		0.609
Detectable light-adapted 3.0 ERG	2 (28.6%)		3 (60.0%)		1 (33.3%)		0.530
(n, %)							
Detectable flicker ERG (n, %)	2 (28.6%)		4 (80.0%)		3 (100%)		0.057
	Right eye	Left eye	Right eye	Left eye	Right eye	Left eye	*p*
BCVA (logMAR)	0.87 ± 0.06	0.91 ± 0.06	1.00 ± 0.10	0.93 ± 0.11	0.80 ± 0.20	0.80 ± 0.2	0.493
CRT (µm)	215.3 ± 16.6	225.7 ± 13.4	220.4 ± 25	211.6 ± 25.7	274 ± 15	197.3 ± 18.5	0.803
MS (dB)	13.4 ± 1.5	15 ± 1.5	15.6 ± 1.5	15.9 ± 1.7	14.4 ± 3	14.4 ± 3.2	0.371
Dark-adapted 0.01 ERG	136.9 ± 11.9	159.9 ± 21.1	151.3 ± 24.2	140.4 ± 27.6	145 ± 27	143.5 ± 23.5	0.515
(b-wave amplitude, µV)							
Light adapted 3.0 ERG	5.7 ± 3.7	9.4 ± 8.3	12 ± 8.7	14.3 ± 11.3	22.6	24.0	0.793
(b-wave amplitude, µV)							
30 Hz Flicker ERG	3.9 ± 2.8	3.3 ± 2.1	3.7 ± 3.2	4.5 ± 3.4	5 ± 0.9	5.1 ± 0.5	0.103
(N1-P1, µV)							

BCVA, best corrected visual acuity; CRT, central retinal thickness; D, diopter; ERG, electroretinogram; MS, macular sensitivity. Data are expressed as mean ± standard error of the mean for continuous variables, and as number of patients (frequency) for categorical variables.

## Data Availability

Data is contained within the article or supplementary material.

## Supplementary Materials

Supplementary materials can be found at https://www.mdpi.com/1422-0067/22/4/1681/s1.

## Abbreviations

ACHM	Achromatopsia
AF	Autofluorescence
BCVA	Best corrected visual acuity
CRT	Central retinal thickness
ERG	Electroretinogram
FAF	Fundus autofluorescence
HGMD	Human Gene Mutation Database
LOVD	Leiden Open Variation Database
MP	Microperimentry
MS	Macular sensitivity
NGS	Next generation sequencing
OCT	Optical coherence tomography
RPE	Retinal pigment epithelium
SNV	Single nucleotide variants
