# Supplementary material for: Clinical and Molecular Characterization of Achromatopsia Patients: A Longitudinal Study
Source: Int J Mol Sci. 2021 Feb 7;22(4):1681. doi: 10.3390/ijms22041681 (PMC7914547; doi:10.3390/ijms22041681)
Supplement: Supplementary file 1 [file ijms-22-01681-s001.pdf]

SUPPLEMENTARY FIGURE

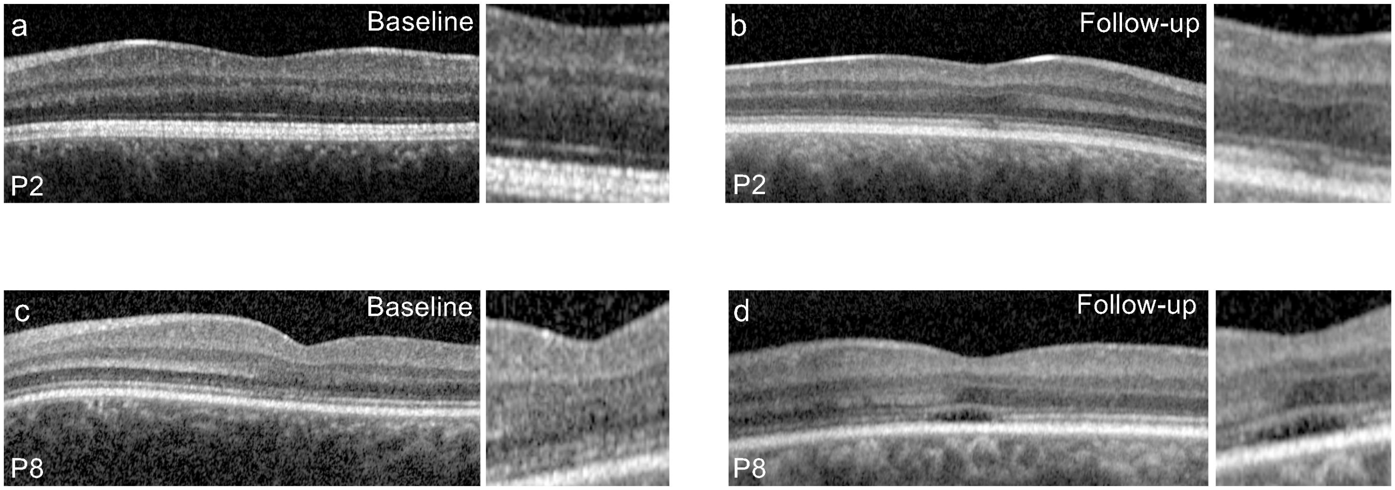

Figure S1 Progression of structural changes in two ACHM patients over the follow-up.
Baseline (**a, c**) and follow-up (**b, d**) SD-OCT images of patients P2 (upper panel) and P8 (lower panel) who progressed to a worse SD-OCT structural grade during the follow-up period. The insets show an enlargement of the corresponding foveal ellipsoid zone. P2 progressed from grade 1 to grade 2, and P8 progressed from grade 2 to grade 3 in the left eye only.

SUPPLEMENTARY TABLE

Table S1 Main clinical findings in the analyzed ACHM cohort at the baseline

**Parameters**	**Study Cohort** (n=21)	
Age (years)	18.0 ± 3.2	
Age at diagnosis (years)	6.6 ± 2.0	
Mean refractive error (D)	+0.16 ± 0.63	
	Right eye	Left eye
BCVA (logMAR)	0.88 ± 0.06	0.88 ± 0.06
CRT (μm)	224.4 ± 9.8	213.9 ± 8.2
MS (dB)	14.6 ± 0.9	15.2 ± 0.9
Dark-adapted 0.01 ERG (b-wave amplitude, μV)	147.2 ± 11.7	163.0 ± 10.9
Light-adapted 3.0 ERG (b-wave amplitude, μV)	12.2 ± 3.5	14.7 ± 7.4
30 Hz Flicker ERG (N1-P1, μV)	5.1 ± 1.2	6.0 ± 1.4

BCVA, Best Corrected Visual Acuity; CRT, Central Retinal Thickness; D, Diopter; ERG, Electroretinogram; MS, Macular Sensitivity

Data are expressed as mean ± standard error of mean
